# Public perceptions of the FDA’s marketing authorization of Vuse on Twitter/X

**DOI:** 10.3389/fpubh.2023.1280658

**Published:** 2023-11-03

**Authors:** Sarah Lee, Zidian Xie, Emily Xu, Yihan Shao, Deborah J. Ossip, Dongmei Li

**Affiliations:** ^1^Goergen Institute for Data Science, University of Rochester, Rochester, NY, United States; ^2^Department of Clinical & Translational Research, University of Rochester Medical Center, Rochester, NY, United States; ^3^Department of Public Health Sciences, University of Rochester Medical Center, Rochester, NY, United States

**Keywords:** e-cigarettes, FDA, authorization, Twitter/X, Vuse

## Abstract

**Introduction:**

On October 12, 2021, the FDA issued its first marketing granted orders for Vuse, the e-cigarette product by R.J. Reynolds Vapor Company. The public perceptions and reactions to the FDA’s Vuse authorization are prevalent on social media platforms such as Twitter/X. We aim to understand public perceptions of the FDA’s Vuse authorization in the US using Twitter/X data.

**Methods:**

Through the Twitter/X streaming API (Application Programming Interface), 3,852 tweets between October 12, 2021, and October 23, 2021, were downloaded using the keyword of Vuse. With the elimination of retweets, irrelevant tweets, and tweets from other countries, the final dataset consisted of 523 relevant tweets from the US. Based on their attitudes toward the FDA authorization on Vuse, these tweets were coded into three major categories: positive, negative, and neutral. These tweets were further manually classified into different categories based on their contents.

**Results:**

There was a large peak on Twitter/X mentioning FDA’s Vuse authorization on October 13, 2021, just after the authorization was announced. Of the 523 US tweets related to FDA’s Vuse authorization, 6.12% (*n*=32) were positive, 26.77% (*n*=140) were negative, and 67.11% (*n*=351) were neutral. In positive tweets, the dominant subcategory was *Cessation Claims* (*n*=18, 56.25%). In negative tweets, the topics *Health Risk* (*n*=43, 30.71%), *Criticize Authorization* (*n*=42, 30.00%), and Big Tobacco (n=40, 38.57%) were the major topics. *News* (*n*=271, 77.21%) was the most prevalent topic among neutral tweets. In addition, tweets with a positive attitude tend to have more likes.

**Discussion:**

Public perceptions and discussions on Twitter/X regarding the FDA’s Vuse authorization in the US showed that Twitter/X users were more likely to show a negative than a positive attitude with a major concern about health risks.

## 1. Introduction

Electronic cigarettes, officially called electronic nicotine delivery system (ENDS) but more commonly known as e-cigarettes, have a growing presence in the American population. Though e-cigarettes were only first introduced to the US market in 2006 as a healthier alternative to traditional cigarettes, their popularity has extended beyond the intended adult smokers (1). A recent 2021 study by the Center for Disease Control and Prevention (CDC) found that e-cigarettes have been the tobacco product of choice for American adolescents since 2014 (2). In 2022 about 3.3% of middle school students and 14.1% of high school students admitted to current e-cigarette use (about 2.55 million in total), with 30.1% of those high school students and 11.7% of those middle school students admitting to using their e-cigarettes daily (3). The rate of e-cigarette use in youth is starkly higher than those of American adults, with only 4.5% of American adults reporting current use of e-cigarettes in 2021 (4).

Though the long-term health effects of e-cigarettes are only beginning to emerge, some symptoms of serious lung disease in people who have used e-cigarettes include cough, trouble breathing, chest pain, nausea, vomiting, diarrhea, fatigue, fever, or weight loss (5–8). A review of pre-clinical and clinical data from different studies determined that e-cigarettes use could have a negative impact on cardiovascular health (9–11). Despite this, a recent survey showed that most current e-cigarette users at least somewhat agree that e-cigarettes are a safe option for smoking cessation as well as safer than traditional and smokeless tobacco (12). As a result, the issue of ENDS products’ position and validity in the American market has become a long battle in public health, but it has now become a legal matter. For any policy related to e-cigarettes, policymakers and public health authorities are trying to balance two public health objectives, preventing the initiation of e-cigarette use in youth or young adult non-smokers and reducing the harm of smoking for smokers through e-cigarette use (13).

On October 12, 2021, the US Food and Drug Administration (FDA) made a landmark decision by announcing the first official marketing authorization of three new ENDS products via the Premarket Tobacco Product Application (PMTA) (14). These grant orders were given to R. J. Reynolds (RJR) Vapor Company for its Vuse Solo e-cigarette device and three accompanying tobacco-flavored e-liquid pods. Given its technology and the results of a study where participants used the approved products, the FDA determined that the Vuse Solo and its accompanying e-liquid pods exposed users to fewer harmful and potentially harmful constituents (HPHCs), which are chemicals found in tobacco products that cause harm to both smokers and non-smokers (15). Further, the FDA assessed the risks and benefits of tobacco product users, non-users, and adolescents before concluding that the potential benefit for smokers drastically reduce or switch from traditional cigarette use outweighs the risk to youth and young adult non-smokers (14). With the FDA PMTA authorization of Vuse, it is important to understand how the public responds to this policy change on e-cigarettes.

Social media platforms such as Twitter (now re-branded as “X”) have become a space for millions of users to post any content of their liking, and these posts have become a unique data source that displays the most current and updated public opinions and discussions. In comparison to other social media sites, Twitter/X data is more accessible and has become a valuable and abundant source. Twitter/X posts (tweets) have previously been used to examine and determine public perceptions of significant public health policies, such as the FDA’s flavor enforcement policy and New York state policy on flavored e-cigarettes (16–19).

In this study, we aimed to understand public perceptions of the FDA’s Vuse authorization using Twitter/X data by examining the attitudes and major topics discussed on Twitter/X. We manually labeled each relevant tweet from the US and categorized them into different attitudes and topics toward the FDA’s Vuse authorization to better understand public perceptions. Our results will better inform future public health policy decisions.

## 2. Materials and methods

### 2.1. Data collection

Following the FDA’s authorization of Vuse on October 12th, 2021, we collected all tweets relating to this authorization between October 12th, 2021, and October 23rd, 2021 through Twitter/X streaming API (Application Programming Interface) using the keyword “Vuse.” A total of 3,852 tweets containing the keyword “Vuse” in either the text or hashtags were collected. After further filtering out retweets and duplicate tweets, we ended up with a dataset comprising 2,356 tweets.

### 2.2. Content analysis of tweets by hand-coding

To understand what might lead to different attitudes towards the FDA authorization of Vuse, we performed a content analysis on these tweets. For content analysis, we adopted the traditional inductive method in this study (20–22). From 2,356 tweets, a random sample of 300 tweets was hand-coded individually by two coders, which were used to develop a codebook (Supplementary Table S1). We only considered tweets that made explicit reference to the FDA’s authorization of Vuse as a policy. We did not consider tweets that simply provided an opinion about any aspect of the Vuse product itself or other e-cigarette products.

All relevant tweets were grouped into three main categories based on the attitude of tweets toward the Vuse authorization announcement: positive attitude, negative attitude, and neutral attitude. All positive tweets were further grouped into four categories: cessation claims, celebration of the authorization, mocking those against the authorization, and other. “Cessation Claims” refers to tweets that expressed support for the FDA authorization of Vuse on the belief that the device would help traditional smokers quit cigarettes and that the device was a healthier alternative to cigarettes. “Celebration of the Authorization” refers to tweets that simply expressed a positive opinion or reaction to the news of Vuse’s authorization. “Mocking Those Against the Authorization” is a category for tweets that not only expressed a positive reaction to the FDA authorization, but also mocked or made fun of other people/institutions that were vocal about their opposition. The positive category “Other” was reserved for tweets that expressed a positive attitude towards the authorization but did not provide an explicit reason. Many of these tweets used positive emoticons to express their support.

All negative tweets were grouped into five categories: health risk, criticize the authorization, complain about tobacco-flavored Vuse products, big tobacco, and other. “Health Risk” is a category of tweets that explicitly expressed concern for the impact on public health as a result of the FDA authorization of Vuse. “Criticize Authorization” refers to tweets that explicitly criticized or expressed disappointment about the FDA’s decision to authorize the sale of Vuse. “Complain about tobacco-flavored Vuse products” includes the complain that only tobacco flavor is available for Vuse and Vuse is an outdated product. “Big Tobacco” tweets explicitly drew a connection between Vuse’s FDA authorization and the big tobacco industry, criticizing this potential conflict of interest. The negative “Other” category was reserved for tweets that expressed a negative attitude towards the authorization but did not provide an explicit reason. Many of these tweets used negative emoticons to express their criticism.

All neutral tweets were grouped into four categories: news, product safety claims, news on specific policies, or other. Tweets that fell into the “News” category were tweets of news article headlines or links that simply stated the fact that the FDA had authorized Vuse in the US market. Tweets under “FDA Claims About Product Safety” simply stated reasons the FDA cited for their decision to authorize Vuse. “Specific Policies” is a category for tweets that explicitly mentioned specific policies and product applications that contributed to the final FDA decision. The neutral “Other” category was reserved for tweets that did not fit into any of the previous neutral categories, in addition to not expressing a personal opinion or attitude towards the authorization.

For the first 300 sample tweets, the kappa statistic between the two coders was 0.91, indicating a high level of agreement. Any differences between the two coders were resolved through discussion by a group of four team members. The remaining 2,056 tweets were single coded by two coders.

### 2.3. Statistical analysis

We calculated the proportion of tweets with different attitudes toward the Vuse authorization, and their differences were tested using the two-proportional *Z*-test with a significant level at 5%. Within each attitude category, we also calculated the distribution of topics. We compared the average (with standard deviation) and median (with interquartile range) number of favorites (likes) of tweets for each attitude category and their respective topics.

## 3. Results

### 3.1. Attitudes towards the FDA authorization of Vuse on Twitter/X

**Figure 1:**
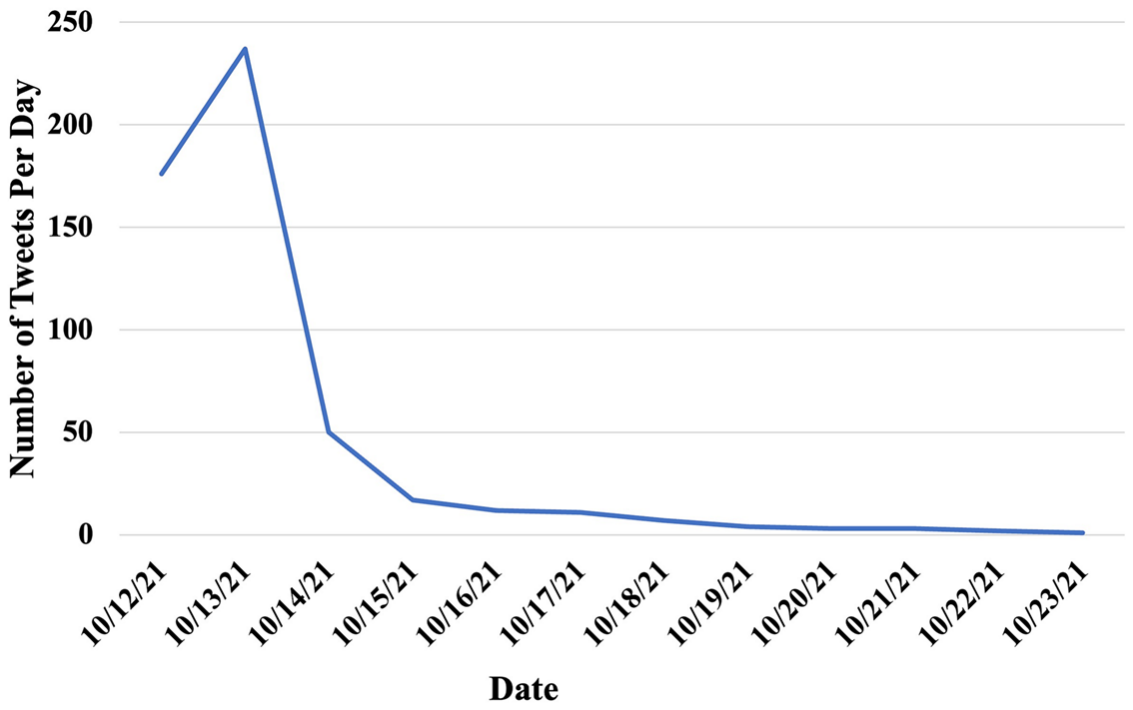
The longitudinal mentions of FDA’s marketing authorization of Vuse on Twitter/X.

From the 2,356 tweets we collected between October 12 and 23, 2022 using “Vuse” as the keyword, only 997 tweets were relevant to the FDA’s authorization of Vuse. Of those 997 tweets, 523 tweets were posted by US Twitter/X users. Among these 523 tweets, 32 tweets (6.12%) showed a positive attitude towards the authorization, 140 tweets (26.77%) showed a negative attitude, and the remaining 351 tweets (67.11%) showed a neutral attitude (Table 1). The proportion of negative tweets was significantly higher than that of positive tweets (*p* < 0.0001). Figure 1 showed the distribution of relevant tweets between October 12th, 2022, and October 23rd, 2022. There was a peak on October 13th, 2022, with 237 tweets, which quickly decreased afterward.

**Table 1:** Topics in tweets related to FDA’s marketing authorization of Vuse.

Attitude towards the Vuse authorization (*n*, %)	Topics	Number of tweets (%)	Average number of likes (SD)	Median number of likes (IQR)
Positive (32, 6.12%)	Total	32 (100%)	9.56 (10.12)	6.5 (14)
	Cessation claims	18 (56.25%)	7.11 (8.07)	3 (12.5)
	Celebrate authorization	6 (18.75%)	7 (12.25)	1 (6.75)
	Mock those against authorization	5 (15.63%)	18.4 (5.9)	15 (7)
	Other	3 (9.38%)	14.67 (16.8)	11 (16.5)
Negative (140, 26.77%)	Total	140 (100%)	5.21 (13.15)	1 (4)
	Health risk	43 (30.71%)	5.19 (11.66)	1 (3)
	Criticize authorization	29 (20.71%)	7.82 (20.8)	1 (4)
	Complain about tobacco-flavored Vuse products	13 (9.28%)	5.69 (8.06)	4 (4)
	Big tobacco	40 (28.57%)	4.08 (11)	0 (2)
	Other	15 (10.71%)	2.87 (4.75)	0 (3.5)
Neutral (351, 67.11%)	Total	351 (100%)	3.62 (20.05)	0 (1)
	News	271 (77.21%)	2.54 (12.73)	0 (1)
	Claims about product safety	67 (19.09%)	5.90 (36.42)	0 (1.5)
	Specific policies	6 (1.71%)	23.67 (33.7)	4 (37.25)
	Other	7 (1.99%)	6.71 (10.01)	0 (11)

SD, standard deviation; IQR, interquartile range.

### 3.2. Topics in tweets related to the FDA authorization of Vuse

**Figure 2:**
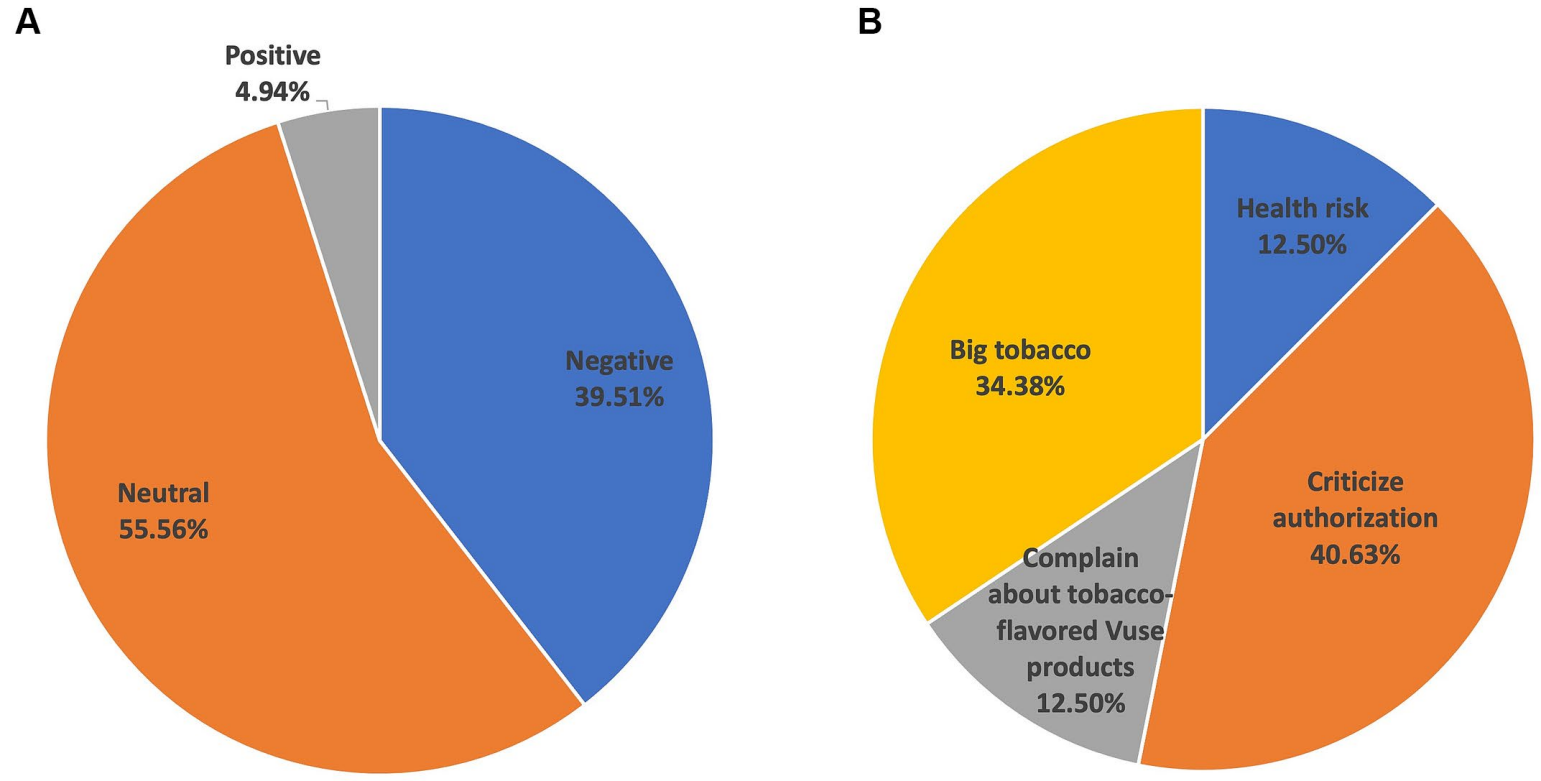
Topics in tweets related to FDA’s marketing authorization of Vuse from October 14th, 2022 to October 23rd, 2022. **(A)** The proportion of tweets with different attitudes. **(B)** The proportion of topics in negative tweets.

As shown in Table 1, among positive tweets, the most popular topic was *Cessation Claims* (*n* = 18, 56.25%), followed by *Celebrate Authorization* (*n* = 6, 18.75%), *Mock Those Against Authorization* (*n* = 5, 15.63%), and *Other* (*n* = 3, 9.38%). Among negative tweets, *Health Risk* (*n* = 43, 30.71%) and *Big Tobacco* (*n* = 40, 28.57%) were relatively popular, followed by *Criticize Authorization* (*n* = 29, 20.71%), Complain about tobacco-flavored Vuse products (*n* = 13, 9.28%), and *Other* (*n* = 15, 10.71%). *News* (*n* = 271, 77.21%) was the dominant topic in neutral tweets. Other neutral topics were less popular, such as *Claims About Product Safety* (*n* = 67, 19.09%), *Specific Policies* (*n* = 6, 1.71%), and *Other* (*n* = 7, 1.99%). Two days (since October 14th, 2022) after the announcement of Vuse authorization, the proportion of negative tweets increased (39.51%, 32/81). Among negative tweets, the proportion of “Criticize authorization” increased from 20.71% to 40.63% (Figure 2).

To examine how each tweet was viewed by other Twitter/X users, we examined the number of likes each tweet received. For positive tweets, the category *Mock Those Against Authorization* had the most likes (Table 1). For negative tweets, all four topics had a similar number of likes, though *Criticize Authorization* had the largest average (7.17) (Table 1). For neutral tweets, it is notable that *News* did not generate as many reactions as *Specific Policies* (Table 1). Table 1 also showed the median and IQR of the number of likes for each category: positive (median = 6.50, Interquartile Range (IQR) = 14), negative (median = 1, IQR = 4), and neutral (median = 0, IQR = 1).

## 4. Discussion

In this study, we examined public perceptions of the FDA’s Vuse authorization by analyzing Twitter/X data. To our knowledge, this is the first study revealing how the public on social media perceived the Vuse authorization. Although most tweets were neutral, there were significantly more negative tweets than positive ones. The major reason for the positive attitude was that the Vuse authorization could help with smoking cessation. In contrast, the concern about health risks associated with vaping and the big tobacco company behind Vuse were the major causes for the negative attitude toward the FDA’s Vuse authorization.

With the increasing popularity of e-cigarettes especially among youth, more concerns focused on the health risks associated with e-cigarettes. With the long-standing debate on if e-cigarettes are a safer alternative to combustible cigarettes, several studies have shown that e-cigarettes have relatively lower health risks than combustible cigarettes (23–25). In addition, aside from the gateway effect of e-cigarettes for cigarette smoking (26, 27), some studies have shown that vaping is considered an effective smoking cessation approach (28–30). In this study, we noticed that among positive tweets toward the Vuse authorization, the predominant theme was the discussion of lower health risks of e-cigarettes and their potential contribution to smoking cessation. This highlights a prevalent belief among certain Twitter/X users regarding the harm reduction benefits of e-cigarettes.

In this study, we observed that there were more tweets with a negative attitude than those with a positive attitude toward the Vuse authorization. In the category of *Health Risk*, Twitter/X users were concerned about health risks or the addictiveness of e-cigarettes as well as the unexpected e-cigarette use for those non-smokers especially among youth as a consequence of the Vuse authorization. The category *Complain about tobacco-flavored Vuse products* mainly complained about the lack of other flavors for approved Vuse products, or the company and product being authorized. Many were confused about why Vuse was being authorized, voicing that the Vuse Solo was an outdated e-cigarette product. The tweets in the *Big Tobacco* category expressed a notion that there was a corrupt deal between the FDA and RJ Reynolds due to the FDA’s “loyalty to Big Tobacco company.” Furthermore, tweets in the *Big Tobacco* category also wondered if the FDA cared about people using vaping as a means of smoking cessation, they would have chosen products that have lower nicotine content and that come from actual vaping companies. In addition, we observed that two days after the announcement of the Vuse authorization, the number of tweets discussing this policy dropped quickly, indicating that the public attention to this policy diminished quickly on Twitter. Interestingly, among those tweets, the proportion of negative tweets, especially those criticizing the Vuse authorization, increased significantly, which suggests that public perceptions of the Vuse authorization were evolving over time. Together, these tweets reflected that many Twitter/X users held a negative attitude toward the Vuse authorization because they were concerned about the health risks of e-cigarettes as well as the intention of authorizing Vuse products.

There were several limitations in this study. There were some challenges in determining which tweets were from US users. The user location feature is not always accurate, with some tweets or Twitter/X users not providing their location information or providing information unrelated to the location. Therefore, some tweets may not be accounted for since the user’s location was not explicitly labeled as the US, which could introduce some biases. While we were trying to follow the best practice for category classification, we can not completely avoid some bias in this process. In addition, the sample size is relatively small in this study, which might limit the generalization of our findings. Moreover, the demographic composition of Twitter/X users, especially Twitter/X users who tweeted about this Vuse authorization, may not be the same as the US population. Therefore, our results may not accurately represent the attitudes of the overall US population. Lastly, since Twitter/X does not provide the demographics of Twitter users, we could not examine the responses to the Vuse authorization between different demographic groups (especially the adolescents), which need to be addressed in future work. How the Vuse authorization affected user behavior remains to be determined in future studies.

## 5. Conclusion

By mining Twitter/X data, we examined public perceptions and discussions regarding the FDA’s Vuse authorization in the US. We demonstrated that more tweets expressed a negative attitude toward the authorization than those with a positive attitude. Understanding how the public perceived and discussed the Vuse authorization could shed light on compliance with the authorization and potential changes in e-cigarette product use, which could help with future regulation of e-cigarette products.

## Data availability statement

The Twitter/X data analyzed for this study are available upon request from the corresponding author.

## Ethics statement

The studies involving humans were approved by this study has been reviewed and approved by the Office for Human Subject Protection Research Subjects Review Board (RSRB) at the University of Rochester (Study ID: STUDY00006570). The studies were conducted in accordance with the local legislation and institutional requirements. Written informed consent for participation was not required from the participants or the participants’ legal guardians/next of kin in accordance with the national legislation and institutional requirements.

## Author contributions

SL: Formal analysis, Visualization, Writing – original draft, Writing – review & editing. ZX: Conceptualization, Data curation, Investigation, Methodology, Supervision, Writing – original draft, Writing – review & editing. EX: Formal analysis, Visualization, Writing – original draft, Writing – review & editing. YS: Data curation, Writing – original draft, Writing – review & editing. DO: Writing – original draft, Writing – review & editing. DL: Conceptualization, Funding acquisition, Methodology, Supervision, Writing – original draft, Writing – review & editing.
