# Supplementary material for: Public perceptions of the FDA’s marketing authorization of Vuse on Twitter/X
Source: Front Public Health. 2023 Nov 3;11:1280658. doi: 10.3389/fpubh.2023.1280658 (PMC10654997; doi:10.3389/fpubh.2023.1280658)
Supplement: Supplementary file 1 [file Table_1.DOCX]

Supplementary Material

**Supplemental Table 1. The codebook for content analysis of tweets.**

| **Attitudes** | **Topics** | **Description** | **Example Tweets** |
| --- | --- | --- | --- |
| Positive | Cessation Claims | Tweets that express the belief that Vuse’s FDA authorization will aid in consumers quitting smoking, reduce harm for those with tobacco addiction, or any other positive outcomes regarding the health of Vuse users. | “The Vuse e-cig FDA authorized isn't popular with kids, and in randomized control trials e-cigs consistently outperform nicotine replacement therapy for effectiveness in helping smokers quit. Oregon's adult smokers deserve access to safer alternatives.”  “FDA approves e-cigarettes by Vuse! A step towards harm-reduction approach to address tobacco addiction/dependence/use disorder. Something, the field has mainly used for other drugs. Don't know the science behind this approach for tobacco, but seems intuitively sensible.” |
|  | Celebrate Authorization | Tweets that express a positive attitude regarding news about Vuse authorization. | “Vuse Solo has received the first of its kind U.S. Food and Drug Administration marketing authorisation for vapour products. This is a key regulatory milestone in BAT’s journey towards delivering #ABetterTomorrow”  “ @US_FDA has made a brilliant, courageous decision.  #ecigarette approval is important as alternative to combustible tobacco. We must stop ordering Americans to do this & don't do that. The hysteria over vaping is part of the prohibitionist mentality in US.” |
|  | Mock Those Against Authorization | Tweet mocks other users/institutions who oppose the FDA authorization, expressing enjoyment of a “win” for those in support of Vuse. | “So the FDA has granted marketing approval for the maker of Vuse, which also makes Camel and Newport The Vuse ecig was also listed as the 2nd most popular brand of ecigs with #teens in the national tobacco youth survey.  @ParentsvsVape and @TobaccoFreeKids must be proud.”  “While everyone is mulling over the FDA's authorization of RAI's Vuse Solo, can we all take a moment and ask for a wellness check? Has anyone checked on our friends at PAVE? I understand they must be shocked.” |
|  | Other | Tweets that express a positive attitude without providing a reason, express support but don’t explicitly fit into the other categories, or simply use positive emoticons or hashtags to express the opinion. | “Reading rhe headline I thought "oh, OK, good" F.D.A. Authorizes E-Cigarettes to Stay on U.S. Market for the First…”  “E-cigarettes are safe, and now that the FDA recognised it in the case of one company, the trend should get replicated across the board, regardless of size and standing” |
| Negative | Health Risk | Tweets that express public health concern towards Vuse’s FDA authorization. | “It amazes me that #ecigs like #Vuse with EXTREMELY high salt based nicotine was ever approved by the @FDATobacco.  Salt nic was developed specifically to make it more #addictive like the ammonia in combustible #tobacco.  There's a big diff. between salt nic and freebase nic.”  “In the wake of the @US_FDA decision, the tobacco industry's marketing machine is undoubtedly acting with unprecedented speed to acquire new customers—smokers or not. FDA—and Congress—must act with equal urgency to protect public health.” |
|  | Criticize Authorization | Tweets that explicitly criticize or express disappointment in FDA’s authorization of Vuse. | “Make it make sense: The FDA gives an ok to VUSE e-cigarettes, its first vape authorization EVER, during a pandemic that attacks lungs?? #FDA #Vape #Vuse”  “On Tuesday, the FDA authorized the legal marketing of e-cigarettes on the US market for the first time. In his Expert Column, Eric Lindblom explains why the FDA’s decision to authorize the sale of RJ Reynolds’ Vuse e-cigarettes is flawed.” |
|  | Complain about tobacco-flavored Vuse products | Tweets that complain about the unavailability of other flavors except tobacco flavor in Vuse products, as well as Vuse is an outdated products. | “What a joke, no one wants to vape a tobacco flavored vuse”  “@MsE_HHS You do realize Vuse has no flavors other than tobacco and was just approved by @FDATobacco” |
|  | Big Tobacco | Tweets that explicitly point out and criticize the connection between big tobacco and Vuse’s FDA authorization. | “The vaping scam continues thanks to the federal government's loyalty to the tobacco industry.”  “Why am I not surprised it’s Vuse. Not only is it the WORST vape on the market, but it’s the one owned by Phillip Morris. This just PROVES that the FDA is in the pockets of Big Tobacco, which is a direct conflict of interest.” |
|  | Other | Tweets that express a negative attitude without providing a reason, express criticism but does not explicitly fit into the other categories, or simply use negative emoticons or hashtags to express the opinion. | “Wait... they did WHAT?”  “Why? 🤬”  “FDA authorizes an e-cigarette for first time, saying benefit for smokers outweighs risk to youths. **Hmm 🤔**” |
| Neutral | News | Tweets that simply are news article titles stating the fact that the FDA has authorized Vuse. | “F.D.A. Authorizes E-Cigarettes to Stay on U.S. Market for the First Time”  “For the first time in history, the FDA authorized three e-cigarette products: the Vuse Solo device and its pods” |
|  | FDA Claims About Product Safety | Tweets that state the FDA’s reasons for approving Vuse. | “FDA authorizes RJReynolds' Vuse e-cigarette and 2 tobacco-flavored cartridges, saying benefit for adult smokers as a smoking cessation device outweighs risk to minor consumers”  “The @US_FDA for the first time on Tuesday authorized an electronic cigarette to be sold in the US. The FDA determined that the potential benefit to smokers who switch completely or significantly reduce their cigarette use would outweigh the risk to youth.” |
|  | Specific Policies | Tweets that explicitly discuss specific policies that contributed to the FDA authorization of Vuse. | “The marketing granted order marks the first for e-cigarette products under the premarket tobacco product application (PMTA) pathway, the @US_FDA said.”  “You could read the FDA guidance to see what they expect to see in a PMTA. The Vuse application referenced its use to cigarette smoking as well as other marketed vapor products.” |
|  | Other | Tweets that don’t express or reveal a valenced opinion on the topic of FDA’s authorization of Vuse. | “Big stock market reaction to the authorization of Vuse Solo in tobacco flavor.”  “I did not see this one coming. Let's see how this goes....” |
